# Supplementary material for: Chondroitin Sulfate and Fucosylated Chondroitin Sulfate as Stimulators of Hematopoiesis in Cyclophosphamide-Induced Mice
Source: Pharmaceuticals (Basel). 2021 Oct 24;14(11):1074. doi: 10.3390/ph14111074 (PMC8623974; doi:10.3390/ph14111074)
Supplement: Supplementary file 1 [file pharmaceuticals-14-01074-s001.zip › pharmaceuticals-1367302-supplementary.pdf]

Supplementary

Chondroitin sulfate and fucosylated chondroitin sulfate as stimulators of hematopoiesis in cyclophosphamide-induced mice

Nadezhda E. Ustyuzhanina, Natalia Yu. Anisimova, Maria I. Bilan, Fedor V. Donenko, Galina E. Morozevich, Dmitry V. Yashunsky, Anatolii I. Usov, Nara G. Siminyan, Kirill I. Kirgisov, Svetlana R. Varfolomeeva, Mikhail V. Kiselevskiy and Nikolay E. Nifantiev

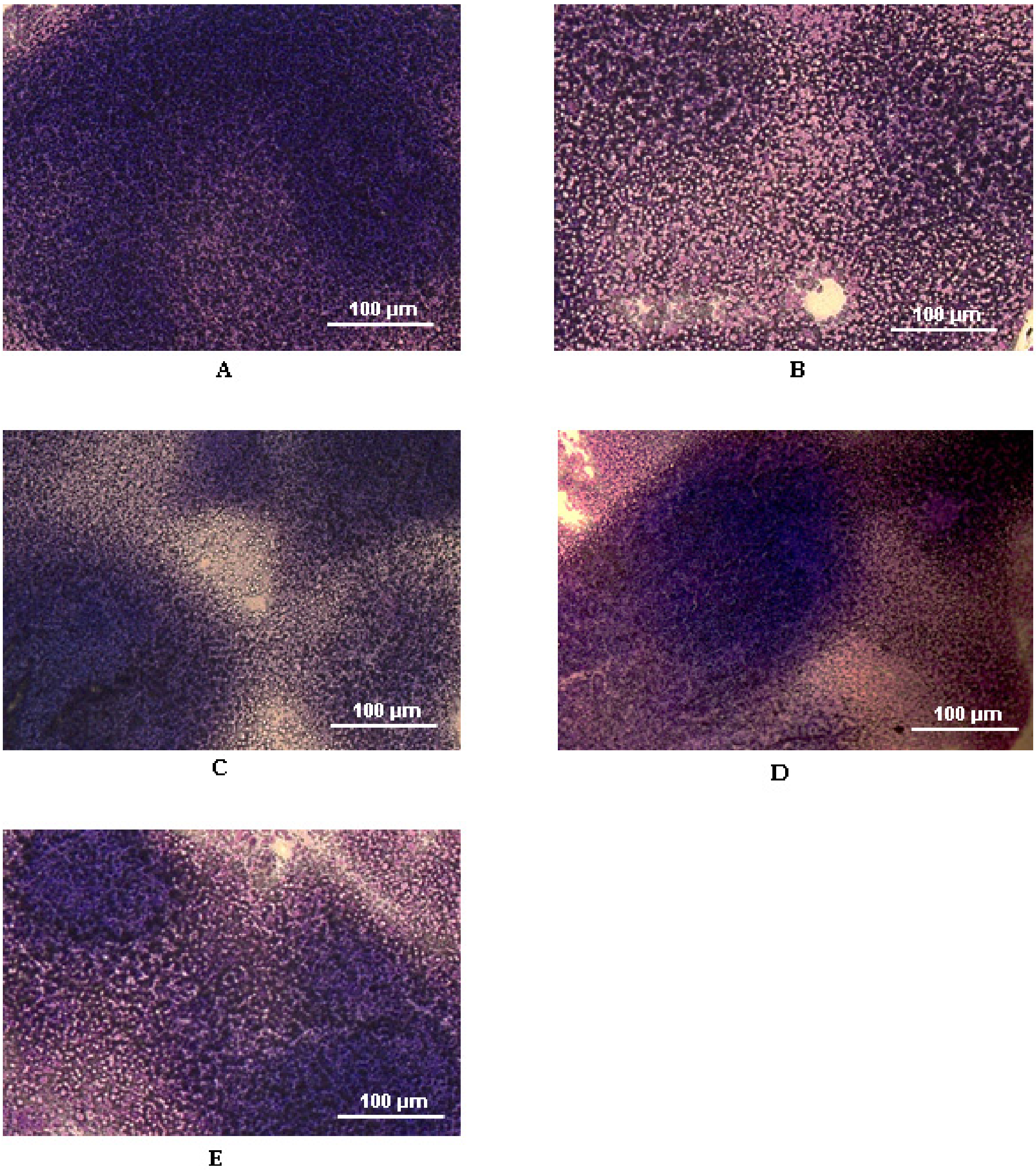

Figure S1. Spleen of mice with CPh-induced immunosuppression after treatment with the tested compounds. (**A**) Control, (**B**) CPh, (**C**) CPh-r G-CSF, (**D**) CPh-**CS**, (**E**) CPh-**FCS**. Hematoxylin-eosin staining of formalin-fixed, paraffin-embedded mouse spleen sections.

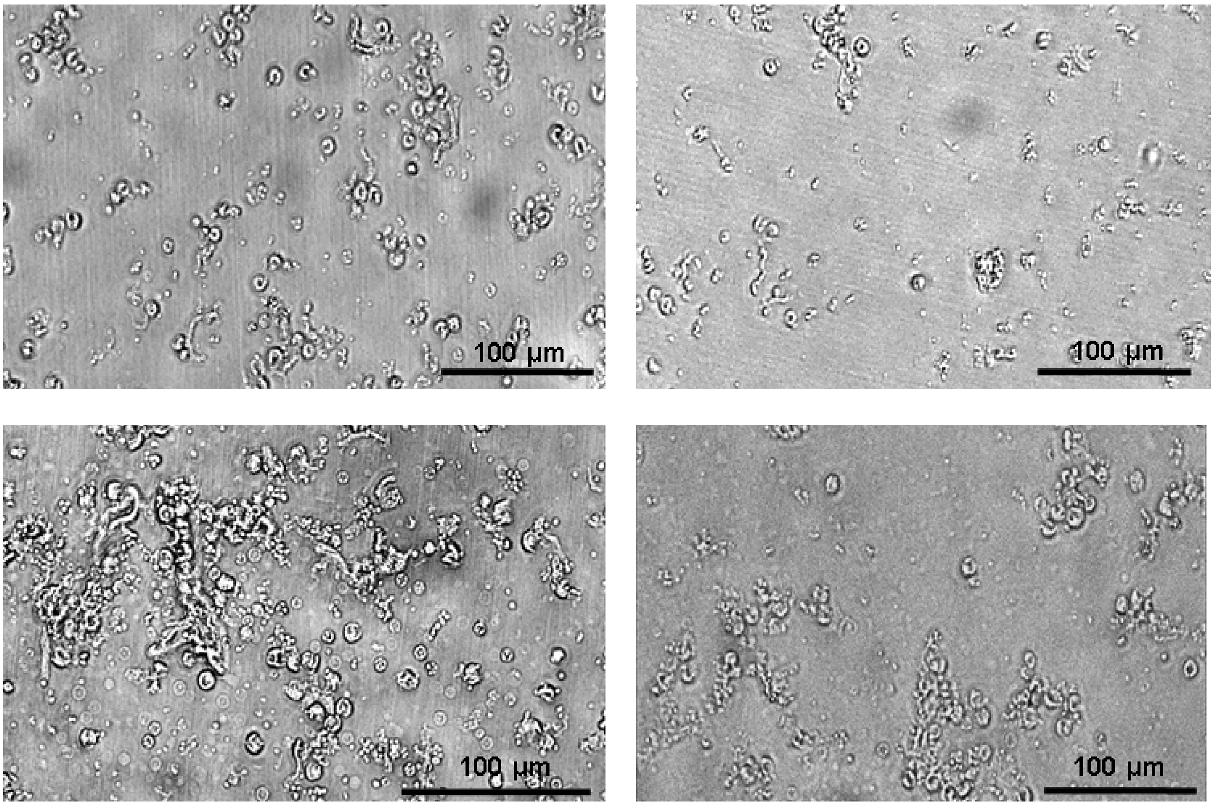

Figure S2. Formation of cell clusters in a culture of bone marrow cells. (**A**) Intact control, (**B**) Cisplatin exposure, (**C**) Cis + **CS**, (**D**) Cis + **FCS**.
